# Accuracy of Intraarticular Injections: Blind vs. Image Guided Techniques—A Review of Literature

**DOI:** 10.3390/jfmk8030093

**Published:** 2023-06-29

**Authors:** Prasenjit Saha, Matthew Smith, Khalid Hasan

**Affiliations:** 1 Department of Medicine, SUNY Upstate Medical University, Syracuse, NY 13210, USA; 2 Department of Orthopaedic Surgery, Virginia Commonwealth University, Richmond, VA 23298, USA

**Keywords:** intra-articular injections, joints, accuracy, blind, image-guided

## Abstract

Intra-articular injections are widely used for diagnostic and therapeutic purposes of joint pathologies throughout the body. These injections can be performed blind by utilizing anatomical landmarks or with the use of imaging modalities to directly visualize the joint space during injections. This review of the literature aims to comprehensively identify differences in the accuracy of intra-articular injections via palpation vs. image guidance in the most commonly injected joints in the upper and lower extremities. To our knowledge, there are no such comprehensive reviews available. A narrative literature review was performed using PubMed and Google Scholar databases to identify studies focusing on the accuracy of blind or image-guided intra-articular injections for each joint. A total of 75 articles was included in this review, with blind and image-guided strategies being discussed for the most commonly injected joints of the upper and lower extremities. Varying ranges of accuracy with blind and image-guided modalities were found throughout the literature, though an improvement in accuracy was seen in nearly all joints when using image guidance. Differences are pronounced, particularly in deep joints such as the hip or in the small joints such as those in the hand or foot. Image guidance is a useful adjunct for most intra-articular injections, if available. Though there is an increase in accuracy in nearly all joints, minor differences in accuracy seen in large, easily accessed joints, such as the knee, may not warrant image guidance.

## 1. Introduction

Intra-articular injections (IAIs) are widely used for various joint pathologies in orthopedics, rheumatology, and sports medicine [1]. Most commonly, IA joint injections are used when conservative management has been unsuccessful in the management of both inflammatory and non-inflammatory intraarticular conditions [2]. They may help diagnose, provide pain relief, reduce inflammation, improve mobility, or even cure the condition. The frequency and dose of the injections are guided by the agent used, goal of management, and disease prognosis [3].

There are various IA agents, with the most common being corticosteroids, local anesthetics, hyaluronic acid, and platelet-rich plasma [1,4]. Corticosteroids supply both immunosuppressive and anti-inflammatory effects, and as such, they can provide an effective treatment of osteoarthritis [4,5]. Local anesthetics are often used as adjuncts to IA corticosteroids for rapid pain relief, which can also be diagnostic [3]. Hyaluronic acid is a glycosaminoglycan that increases the viscosity and elasticity of synovial fluid to reduce further stress on weakened articular cartilage [4,6]. Finally, platelet-rich plasma aids in the treatment by delivering growth factors and bioactive molecules directly to the injured site [4].

Traditionally, IA injections are performed blindly, solely using physical examination and guidance from anatomical landmarks. With proper training, this method can be performed efficiently in a clinic setting with few resources [7]. However, the injection path and joint space are not directly visualized, leaving the potential for damage to surrounding structures and difficulty in accessing the joint with relatively small variations in the anatomy [2,7]. Various imaging modalities are available to use during IAIs, including fluoroscopy, ultrasound, computed tomography (CT), and magnetic resonance imaging (MRI), although these methods vary greatly in their ease of use and access (Table 1) [8]. Fluoroscopy is becoming more readily available and allows for the visualization of the joint space with minimal ionizing radiation [7]. Furthermore, a contrast may be used to improve the efficiency of the IAI under fluoroscopy, though it is not often used by practitioners outside of radiology [1]. Ultrasound is readily available, efficient, and emits no ionizing radiation. Additionally, the separation between the clinician’s needle and important soft tissue structures, such as neurovascular bundles, can be visualized in real time [2]. CT and MRI offer enhanced soft tissue visualization and multiplanar views. However, both are more expensive, time-consuming, and not as easily accessible, reserving these methods for more complicated or higher risk IAIs [1,9,10,11,12,13].

For an IAI to effectively achieve diagnostic and therapeutic goals, clinicians must be able to accurately infiltrate the joint of interest. Injections that miss the joint may lead to false diagnoses and decreased substrate effect. In order to identify relevant studies, a narrative review of the literature was performed using PubMed and Google Scholar databases. Search words included “accuracy”, “intra-articular injection”, “image-guidance”, “blind”, “ultrasound”, “fluoroscopy” together with each joint discussed below. Randomized control trials and systematic reviews were used where available. Only studies that analyzed accuracy published after 2000, were performed exclusively on humans, and were originally printed in English were selected. We did not include return to sports as there is a lack of data in several joints analyzed in this review and athletes are a relatively small population outside of sports medicine. The purpose of this review is to comprehensively synthesize current literature on the accuracy of the various methods of IAIs with and without imaging guidance throughout the body.

## 2. Upper Extremity

### 2.1. Shoulder

An IAI of the shoulder may target the glenohumeral, acromioclavicular (AC), or subacromial joints for various conditions [1]. The three approaches for glenohumeral IAIs include the anterior, rotator interval, and posterior approaches. In the anterior approach, the needle is injected lateral to the coracoid process toward the space between the middle and inferior thirds of the medial humeral head. The rotator interval approach involves the insertion of the needle into the superomedial quadrant of the humeral head to enter the space between the subscapularis and supraspinatus tendon. In the posterior approach, the needle is inserted into the inferomedial quadrant of the humeral head. When imaging is available, fluoroscopy is primarily used for the anterior and rotator interval approaches, while ultrasound is generally used for the posterior approach [1,14]. In an AC joint injection, the needle is inserted superiorly and anteriorly into the depression at the end of the distal clavicle. For a subacromial joint injection, the needle is advanced posterolateral after palpating the edges of the acromion [15].

Accuracy of blind shoulder IAIs is variable in the literature, though higher accuracy rates are consistently reported with image guidance [16]. Recent studies have shown high accuracy rates with the blind anterior approach at 94% and the posterior approach at 78% [17]. Multiple systemic reviews have been performed to compare blind and imaging-guided shoulder IAIs. In 2011, Daley et al. evaluated 12 studies and found that the average accuracy of a blind glenohumeral joint IAI was 79% (range of 27–100%) versus 95% (range of 83–100%) with image guidance, which included fluoroscopy, ultrasound, and MRI. In seven studies with a subacromial IAI, the accuracy rate for a blind IAI was 72% (range 29–100%) compared to 100% with image guidance. In two studies on AC joint injections, the average accuracy of the blind IAI was 45% (range 38–55%) compared to 100% with image guidance [18]. In 2015, Aly et al. found in their systematic review that the accuracy rate of the blind glenohumeral joint IAI was 72.5% versus 92.5% with ultrasound guidance. For a subacromial IAI, the difference between the accuracy rate of blind anatomic guidance versus ultrasound guidance was not statistically significant with reported rates of 70% versus 65%. For AC joint IAIs, the accuracy rate of blind injections was 68.2% versus 93.6% with ultrasound guidance [19]. In 2017, Simoni et al. evaluated 13 studies and found that the accuracy rate of blind shoulder glenohumeral IAIs varied from 42% to 100% while also comparing the accuracy based on the imaging method used. In four of these studies, fluoroscopic guidance provided accuracy ranging from 97 to 100% for glenohumeral IAIs whereas six articles using ultrasound guidance showed accuracy ranging from 92 to 100%. Finally, in five of these articles, MRI guidance produced an accuracy rate ranging from 96.8% to 100% [20].

### 2.2. Elbow

Elbow joint IAIs are primarily performed using a lateral or posterior approach. In the more commonly used lateral approach, the triangle formed by the olecranon, radial head, and lateral epicondyle is used as an anatomic landmark, with the needle oriented anteriorly toward the radiocapitellar joint [1,14,21]. In the posterior approach, the space between the medial epicondyle and olecranon is used to aim at the olecranon fossa [1]. Elbow IAI injections that use image guidance most commonly use ultrasound [22]. Although fluoroscopy is commonly used for a contrast injection prior to MR arthrography of the elbow, there are few data on fluoroscopy-guided elbow IAI accuracy rates [16].

Elbow joint injections performed blindly have a wide reported range of accuracy compared to ultrasound-guided injections in the literature [16,18]. Lopes et al. found the accuracy rate of blind IAIs of the elbow to be 100% within their study group of rheumatoid patients [23]. Balint et al. compared the joint aspiration rate of blind and ultrasound-guided IAIs by trained rheumatologists, where they found the blind aspiration accuracy rate to be 37.5% versus 100% with ultrasound [24]. Kim et al. reported that the posterior approach for elbow joint injections was accurate 77.5% of the time with blind palpation compared to 100% with ultrasound guidance [25]. Cunnington et al. found the accuracy rate of palpation-guided elbow IAIs to be 64% compared to 91% in the ultrasound-guided elbow IAI [26]. Overall, the accuracy rates range from 37.5 to 100% with blind injections versus 91 to 100% with ultrasound-guided elbow IAIs, leading to the majority of the reviewed literature suggesting routine use of image guidance for elbow injections.

### 2.3. Wrist

IAI injections of the wrist can prove to be complicated due to the small, obliquely oriented joint space and the presence of several joint-spanning ligaments [27]. The traditional method of injection involves a dorsal approach approximately 1 cm distal to Lister’s tubercle between the volarly angled articular surface of the radius and lunate [28]. The alternative ulnocarpal approach can be used with a needle insertion at the proximal ulnar edge of the triquetrum. Finally, a mid-carpal approach can be directed at the junction of the triquetrum, lunate, capitate, and hamate [14].

The accuracy of blind IAI injections of the wrist joint ranges from poor to excellent in the literature. In 1993, Jones et al. performed IA corticosteroid injections of the wrist in eight patients with only 50% being intraarticular [17]. Phillips et al. evaluated the accuracy of blind ulnocarpal IAIs across different training levels and found the rate to be 71% without significant differences between levels of training [28]. In a study of rheumatoid arthritis patients, Lopes et al. found the accuracy rate of blind IAIs of the wrist by trained rheumatologists to be 97% [23].

Several studies in the literature comparing ultrasound guidance with blind wrist IAIs have found no significant difference in accuracy. Cunnington et al., in their study of patients with inflammatory arthritis, found the accuracy rate of ultrasound-guided wrist IAIs to be 79% as compared to 75% in blind, palpation-guided IAIs [26]. Luz et al. studied patients with rheumatoid arthritis and found the accuracy rate of both ultrasound-guided and blind wrist IAIs to be 90% [27]. To et al., in their study, also found no increase in accuracy with image guidance. They reported the accuracy rate for IA RC joint injections to be 95% with blind anatomic guidance versus 85% with ultrasound guidance [29]. Despite the lack of significant differences in the aforementioned literature, the accuracy of blind wrist IAIs is consistently questioned. Manadan et al. compared radiocarpal joint arthrocentesis sites marked by rheumatologists with fluoroscopically identified radiocarpal joint sites to determine accuracy. They found that the marked sites were, on average, 0.85 cm away from the fluoroscopically identified sites, which was determined to be significant. They concluded that fluoroscopic guidance can improve the accuracy of wrist IAIs [30]. Ali et al. found that the accuracy rate for the dorsal radial contrast injection was 100% for ultrasound and fluoroscopy-guided injections compared to a rate of 72% for those done by palpation [31].

### 2.4. Hand

IAI injections in the joints of the hand are regularly used for the diagnosis and treatment of multiple conditions, including osteoarthritis and rheumatoid arthritis. Palpation of anatomic landmarks is traditionally used for IAIs depending on the joint targeted; however, the joints of the hand are much smaller targets when compared to larger peripheral joints. This makes them increasingly susceptible to inaccurate, extraarticular injections when approached blindly [32,33,34].

The accuracy of blind IAIs of the hand has varied in the literature from poor to good, with great variability between different joints. Lopes et al. found the accuracy of 39 blind IAIs of metacarpophalangeal (MCP) joints by trained rheumatologists to be 97% [23]. Mandi et al. found, in patients with CMC osteoarthritis, a blind IAI of the CMC joint was accurate 92% of the time after confirmation by ultrasound [35]. However, Hunter et al. found the collective accuracy of various blind hand joint IAIs, including the thumb and fifth digit CMC joints, MTP joint, ITP joint, and STT joint, to be 32.24%. Furthermore, this study showed that the accuracy was lower in smaller joints of the hand [36]. More recently, Katt et al. evaluated 82 patients with thumb CMC joint arthritis and found the accuracy of blind IAIs to be 80% [37].

Guided injections within the hand have shown improved accuracy in the available literature. Pollard et al. evaluated patients with CMC joint arthritis and found that an IAI with fluoroscopy guidance was accurate 100% of the time as compared to 81.8% with a blind IAI [38]. Karalezli et al. divided patients with trapeziometacarpal osteoarthritis into a fluoroscopy-guided IAI group and a blind IAI group. They found that the blindly injected cohort experienced more pain associated with the injection, thought to be due to a para-articular injection or periosteal injection from decreased accuracy [39]. However, fluoroscopy has several disadvantages when compared to ultrasound, specifically with regard to cost, radiation, and the inability to visualize important soft tissue structures such as neurovascular bundles. In addition, fluoroscopy may require a contrast for adequate visualization of the joint space, particularly for small joints within the hand, which reduces the volume of therapeutic agents that can be used [34]. Ultrasound may be a better option for IAI injections. Umphrey et al. found that ultrasound-guided thumb basal joint injections in 17 cadaveric models had an accuracy rate of 94% [40]. To et al. found the accuracy rate of US-guided IAIs in patients with CMC arthritis to be 72% as compared with 38% in blind IAIs [29]. These studies have consistently shown that regular use of image guidance may be indicated for IAIs of the hand (Table 2).

## 3. Lower Extremity

### 3.1. Hip

The standard approach to hip joint IAIs is an anterior approach with the needle directed toward the lateral aspect of the femoral head and neck junction [1]. The blind hip IAI is complicated by the depth of the joint, the difficulty in palpating anatomic landmarks, and the presence of important neurovascular structures close to the needle’s path [41]. Leopold et al. reported a blind hip IAI accuracy of 60% and 80% for the anterior and lateral approaches, respectively. Leopold et al. also found that 27% of blind hip injections using the anterior approach pierced or made contact with the femoral nerve, highlighting the importance of using imaging guidance to prevent significant complications [42]. Yiannakopoulos et al. found the accuracy of 35 blind hip IAIs to be 94.3%, although they determined the injection point based on a pre-injection hip radiograph using three anatomical reference points: ASIS, the cephalic femoral head/neck junction, and the caudal head/neck junction [43].

Fluoroscopy has traditionally been the imaging modality of choice for the hip, though the complexity of the injection often requires a referral to musculoskeletal radiology, causing delays in care [44]. More recently, the use of ultrasound guidance has increased in popularity [1]. In Sofka’s retrospective review of 358 adults who underwent hip IAIs with ultrasound, there were no cases of neurovascular injury [45]. Hoeber et al. evaluated 136 ultrasound-guided hip IAIs and 295 blind hip IAIs and found the accuracy rate to be 100% and 72%, respectively [46]. Smith et al. found the accuracy rate of 30 ultrasound-guided hip IAIs to be 97% [47]. Balog et al. found a similar accuracy rate of 96% in 50 ultrasound-guided hip IAIs [44]. No studies available in the literature have compared the accuracy of ultrasound vs. fluoroscopy for hip IAIs. Martinez-Martinez et al. found that both ultrasound and fluoroscopy were useful in injecting contrast material for CT and MRI arthroscopy, though they did not report accuracy rates [48]. Byrd et al. studied 50 patients who underwent ultrasound-guided IAIs after previously receiving fluoroscopy-guided IAIs. They found that patients preferred an ultrasound-guided injection as it was less painful and more convenient [49].

### 3.2. Knee

Knee IAIs can be performed through multiple different approaches, with injection sites being divided into lateral and medial approaches. Medial approaches include medial midpatellar (MMP), superomedial patellar (SMP), and anteromedial joint line (AMJL) approaches. Lateral approaches include lateral midpatellar (LMP), superolateral patellar (SLP), lateral suprapatellar bursa (LSB), and infrapatellar (IFP) approaches [50]. Approaches and accuracy vary with different knee pathologies, such as knee osteoarthritis that narrows the medial joint space or patellofemoral space [51].

Multiple systematic reviews have compared the accuracy of blind knee IAIs with imaging-guided IAIs. In 2013, Maricar et al. studied 23 publications that evaluated the accuracy of blind and guided knee IAIs. In studies assessing blind knee IAIs, they found that the accuracy of medial approaches was 82% at the SMP, 74% at the AMJL, and 64% at the MMP site. In the lateral approach, the accuracy rate was 87% at the SLP, 84% at the LMP, 83% at the LSB, and 70% at the ALJL site. In studies assessing image-guided knee IAIs (ultrasound and fluoroscopy), they found that the accuracy rate of medial approaches was 100% at the SMP and 86% at the MMP site. The accuracy of the lateral approaches ranged from 95 to 100% with image guidance. A pool study comparison demonstrated that there were significant improvements in accuracy with image guidance in all injection sites except for the LMP site (*p* = 0.05) [50].

In 2021, Fang et al. studied 12 publications on the accuracy of knee IAIs. They found that blind knee IAIs had an accuracy rate that ranged from 77.3% (midpatellar approach) to 95.74% (superolateral approach). Ultrasound guide knee IAIs had an accuracy rate greater than 95% in all approaches. Across all analyzed studies, ultrasound-guided injections were more accurate than blind injections regardless of the approach used. Furthermore, the accuracy of injections can be improved using approaches where synovial masses are present, as seen in rheumatoid arthritis patients [23,52,53].

However, the blind knee IAI has been shown to be highly accurate, with rates upwards of 90%, with certain approaches performed by experienced clinicians [50,54,55]. Additionally, the data support the theory that the superolateral approach to the knee does not necessarily require expert-level skill or training for an accurate injection [23]. Given the size of the knee joint and the relative ease of IAIs, the added inconvenience and cost of guided knee IAIs may not always be necessary [56].

### 3.3. Foot and Ankle

The joints of the foot and ankle are typically injected via palpation of the anatomic structures, although small joint spaces, complex anatomic shapes, and the close proximity of neurovascular structures may present a challenge to accurate injections [57]. Additionally, because many of the joints within the foot are in close proximity, it is difficult to determine which joint is responsible for the pain, making an accurate injection increasingly important for proper diagnosis [1].

### 3.4. Ankle

The ankle or tibiotalar joint is commonly approached anteriorly with an insertion of the needle between the extensor hallucis longus (EHL) and extensor digitorum longus (EDL) tendon to target the talar dome. Alternatively, the medial or lateral clear space may be targeted. Available studies evaluating blind ankle IAIs have consistently shown poor accuracy. In 1993, Jones et al. reported an accuracy rate of ankle 66.7% for blind ankle IAIs [17]. Lopes et al. reported a blind ankle IAI accuracy rate of 77% in patients with rheumatoid arthritis [23]. Ramesh et al. reported a blind ankle IAI accuracy rate of 63% when performed by orthopedic surgeons with different skill levels, including fellowship-trained foot and ankle specialists [58]. Heidari et al. compared the blind ankle IAI accuracy rates of anteromedial and anterolateral approaches. They found 77% of anteromedial injections were successful as compared to 86.2% of anterolateral injections, although the difference was not significant [59]. Muhammad et al. reported a relatively high accuracy, with a rate of 93% for blind ankle IAIs, though with a small sample size of 14 patients [60].

The addition of imaging modalities has repeatedly shown improved accuracy for ankle IAIs. Cunnington et al. found that the ultrasound-guided ankle IAI was accurate 85% of the time as compared to 58% in blind ankle IAIs [26]. Reach et al. found that 100% of ultrasound-guided ankle IAIs were within the joint space [61]. Wisniewski et al. also reported the accuracy of ultrasound-guided ankle IAIs to be 100% as compared to 85% in blind ankle IAIs [62]. Interestingly, Khosla et al. found an accuracy rate of 100% for both ultrasound and landmark-guided IAIs in their cadaveric study [57]. Stornebrink et al. studied the use of needle arthroscopy in guiding ankle IAIs and found the accuracy rate to be 88%. The remaining 12% where the IAI was unsuccessful had Kellgren–Lawrence grade IV osteoarthritis, indicating that the joint space narrowing from severe osteoarthritis can complicate an ankle IAI [63].

### 3.5. Hindfoot

The most commonly injected joint in the hindfoot is the subtalar joint, which consists of both anterior and posterior components [1]. For subtalar joint IAIs, the anterolateral, posterolateral, or posteromedial approaches are used [64]. Other joints in the hindfoot that may be injected include the talo-navicular (TNJ) and calcaneocuboid (CCJ) joints.

In studies comparing blind and image-guided IAIs of the subtalar joint, a blind injection has been shown to have variable results, though typically inferior joint space infiltration when compared to ultrasound-guided IAIs [65]. Kraus et al., in their cadaveric study, compared the accuracy rates of anterolateral versus posterolateral subtalar injections and found them to be 67.6% and 91.2%, respectively [66]. In the most recent available study, Muhammad et al. found the accuracy of blind subtalar IAIs to be 67% [60]. Kirk et al. studied blind posterior subtalar IAIs and found the accuracy rate to be 97%; however, joint extravasation was seen in 27% of these injections [67]. Wisniewski et al. found the accuracy of blind subtalar IAIs to be 58% vs. 83% in ultrasound-guided injections [62]. Khosla et al. found that ultrasound and palpation-guided subtalar injections’ accuracy rate was 100%, though they did not evaluate the joint extravasation [57]. Smith et al. found 100% accuracy with ultrasound-guided subtalar IAIs using three different approaches; anterolateral, posterolateral, and posteromedial [64]. Reach et al. found that ultrasound-guided subtalar was 90% in a cadaveric model [61].

The limited studies in the literature on TNJ and CCJ IAI accuracy also show improved results with ultrasound guidance. Jha et al., in a cadaveric study on TNJ IAI, found that all ultrasound-guided injections were successful, while all palpation-guided injections were unsuccessful [68]. Reach et al. found the accuracy rates of TNJ and CCJ blind IAIs to be 50% and 60%, respectively [61].

### 3.6. Midfoot

Joints commonly injected for midfoot pathology include the tarsometatarsal (TMT) joints and navicular-cuneiform joints (NCJ). TMT joints can be further divided into medial (first TMT), middle (second and third TMT), and lateral columns (fourth and fifth TMT) [69]. Although there are limited studies in the literature, improved accuracy of IAIs with image guidance has been reported in these relatively small joints. Muhammad et al., in a cadaveric study, found the accuracy rate of blind IAIs of NCJ and TMT joints (unspecified) to be 33% and 50%, respectively [60]. Khosla et al. compared the accuracy rates of palpation vs guided IAIs of the first and second TMT joints and found the accuracy rates of palpation, ultrasound, and fluoroscopy-guided IAIs of the first TMT to be 27.3%, 71.4%, and 85.7%, respectively. Additionally, the accuracy rates for palpation, ultrasound and fluoroscopy-guided IAIs of the second TMT joint were 28.6%, 57.14%, and 92.8%, respectively [57]. Drakonaki et al. studied patients with midfoot joint degenerative disease who received ultrasound-guided IAIs and 78.4% had continued pain relief after 3 months, which indirectly indicates the accuracy of ultrasound-guide midfoot IAIs [70].

### 3.7. Forefoot

Few studies have evaluated the accuracy rates of blind versus image-guided injections of the metatarsophalangeal (MTP) and interphalangeal (ITP) joints of the forefoot. Muhammed et al. reported the accuracy rates of blind injections of the first MTP, lesser MTP, and first interphalangeal joints to be 75%, 63%, and 67%, respectively [60]. Reach et al., in their cadaveric study, found the accuracy rates of ultrasound-guided first and second MTP IAIs to be 100% [61]. Wempe et al. also found the accuracy rate of ultrasound guild first MTP IAIs to be 100% [71] (Table 3).

## 4. Conclusions

In the available literature, image-guided intra-articular injections, particularly ultrasound, have been shown to be more accurate than palpation-guided IAIs for all joints [72]. Drastic increases in accuracy rates with image-guided versus blind IAIs are seen with smaller joints, such as those in the hand and foot, compared to smaller differences in larger peripheral joints, such as the knee [73]. Additionally, image guidance with ultrasound allows for navigation through soft tissue, a distorted anatomy, and neurovascular structures, decreasing complication rates. However, research on the cost-effectiveness and current utilization of image-guided IAIs is limited and inconclusive [74]. The short- and long-term clinical benefits of image-guided vs blind IAIs need to be studied further [8], although there are some data reporting improved outcomes in terms of pain, function, and mobility when image guidance is used, potentially secondary due to increased accuracy [23,37,75]. Regardless, given the improved accuracy rates, image-guided IAIs should have a continually increasing role in the diagnostic and therapeutic management of peripheral joint pathology.

The main limitation of this review was the variation in available data between joints, with large peripheral joints such as the knee being well studied while there were few articles on the small joints of the foot. Further studies on smaller joints including those in the foot would allow for a more accurate representation of the improvement with image guidance. Other limitations include varying methodologies between articles and different approaches utilized with injections. Further studies on the cost-effectiveness of image-guided IAIs would also be helpful to better determine the role of image guidance in the clinical setting.

## Figures and Tables

**Table 1:** Advantages and Disadvantages of Imaging Modalities.

	Advantages	Disadvantages
Blind	Quickest Most inexpensive Minimal setup required	Difficulty with small joints Body habitus affects landmarks Difficult to confirm if intraarticular Often inaccurate
Ultrasound	Inexpensive Non-invasive adjunct No radiation Does not use contrast material Easily visualize joint space Visualize soft tissue structures Immediate assessment post injection	Operator dependent Equipment required Body habitus can obstruct view Longer procedure times
Fluoroscopy	Effective for deep, complex injections Less training than US Allows for contrast material Identifies extravasation Confirmation of placement in the joint May assist with diagnosis in real time	Risks soft tissue structures Ionizing radiation Less available than US often Complex IAI may require a radiologist Longer procedure times

**Table 2:** Upper Extremity Blind vs. Guided Success.

Upper Extremity	Author	Joint	Blind Success Rate	Image Guided Success Rate	Imaging Used	Systematic Review Article?
Shoulder	Rijs [17]	Anterior glenohumeral	94%	-		
	Rijs [17]	Posterior glenohumeral	78%			
	Daley [18]	Subacromial	72%	100%		yes
	Daley [18]	AC joint	45%	100%		
	Aly [19]	Glenohumeral	72.5%	92.50%		yes
	Aly [19]	Subacromial	70%	65%		
	Aly [19]	AC joint	68%	93.60%		
	Simoni [20]	Glenohumeral	-	97–100%	Fluoroscopy	yes
	Simoni [20]	Glenohumeral	-	92–100%	Ultrasound	
	Simoni [20]	Glenohumeral	-	96.8–100%	MRI	
						
Elbow	Jones [17]		83%	-		
	Lopes [23]		100%	-		
	Kim [25]		77%	100%	Ultrasound	
	Cunnington [26]		61%	100%	Ultrasound	
						
Wrist	Jones [17]		50%	-		
	Phillips [28]		71%	-		
	Cunnington [26]		75%	79%	Ultrasound	
	Luz [27]		90%	90%	Ultrasound	
	To [29]		95%	85%	Ultrasound	
	Ali [31]		72%	100%	Ultrasound and Fluoroscopy	
						
Hand	Lopes [23]	MCP	97%	-		
	Mandl [35]	CMC	92%	-		
	Hunter [36]	CMC	63.6%	-		
	Hunter [36]	MCP	83%	-		
	Katt [37]	CMC	80%	-		
	Pollard [38]	CMC	-	100%	Fluoroscopy	
	Umphrey [40]	CMC	-	97%	Ultrasound	
	To [29]	CMC	38%	72%	Ultrasound	

**Table 3:** Lower Extremity: Blind vs. Guided Success.

Lower Extremity	Author	Joint	Blind Success Rate	Image Guided Success	Imaging Used	Systematic Review Article?
Hip	Leopold [42]	Anterior	60%	-		
	Leopold [42]	Posterior	80%	-		
	Yiannakopoulos [43]		94.30%	-		
	Hoeber [46]		72%	100%	Ultrasound	yes
	Smith [47]		-	97%	Ultrasound	yes
	Balog [44]		-	96%		
						
Knee	Maricar [50]	SMP	82%	-		yes
	Maricar [50]	AMJL	74%	-		yes
	Maricar [50]	MMP	64%	-		yes
	Maricar [50]	SLP	87%	-		yes
	Maricar [50]	LMP	84%	-		yes
	Maricar [50]	LSB	83%	-		yes
	Maricar [50]	ALJL	70%	-		yes
	Maricar [50]	SMP	-	100%	Ultrasound and Fluoroscopy	yes
	Maricar [50]	MMP		86%	Ultrasound and Fluoroscopy	yes
	Fang [53]		77.3–95.74%	>95%	Ultrasound	yes
	Hermans [54]	Superolateral	91%	-		yes
	Jackson [55]		93%	-		
						
Ankle	Jones [17]		66.70%	-		
	Lopes [23]		77%	-		
	Ramesh [58]		63%	-		
	Heidari [59]	Anteromedial	77%	-		
	Heidari [59]	Anterolateral	86.20%	-		
	Muhammad [60]		93%	-		
	Cunnington [26]		58%	85%	Ultrasound	
	Reach [61]		-	100%	Ultrasound	
	Kholsla [57]		100%	100%	Ultrasound	
						
Hindfoot	Kraus [66]	Subtalar anterolateral	67.60%	-		
	Kraus [66]	Subtalar posterolateral	91.20%	-		
	Muhammad [60]	Subtalar	67%	-		
	Kirk [67]	Posterior subtalar	97%	-		
	Wisniewski [62]	Subtalar	58%	83%	Ultrasound	
	Khosla [57]	Subtalar	100%	100%	Ultrasound	
	Smith [64]	Subtalar	-	100%	Ultrasound	
	Reach [61]	Subtalar	-	90%	Ultrasound	
	Jha [68]	Talonavicular	0%	100%	Ultrasound	
	Reach [61]	Talonavicular	50%	-		
	Reach [61]	Calcaneocuboid	60%	-		
						
Midfoot	Muhammad [60]	Naviculocuneiform	33%	-		
	Muhammad [60]	Tarsometatarsal	50%	-		
	Khosla [57]	First tarsometatarsal	27.30%	71.4%	Ultrasound	
	Khosla [57]	First tarsometatarsal	27.30%	85.7%	Fluoroscopy	
	Khosla [57]	Second tarsometatarsal	28.60%	57.14% & 92.8%	Ultrasound	
	Khosla [57]	Second tarsometatarsal	28.60%	57.14% & 92.8%	Fluoroscopy	
						
Forefoot	Muhammad [60]	First MTP	75%	-		
	Muhammad [60]	Lesser MTPs	63%	-		
	Muhammad [60]	First interphalangeal	67%	-		
	Reach [61]	First and second MTP	-	100%	Ultrasound	
	Wempe [71]	First MTP	-	100%	Ultrasound	

## Data Availability

Data sharing not applicable.
